# Nascent SecM Chain Outside the Ribosome Reinforces Translation Arrest

**DOI:** 10.1371/journal.pone.0122017

**Published:** 2015-03-25

**Authors:** Zhuohao Yang, Ryo Iizuka, Takashi Funatsu

**Affiliations:** Laboratory of Bio-analytical Chemistry, Graduate School of Pharmaceutical Sciences, The University of Tokyo, Tokyo, Japan; University of British Columbia, CANADA

## Abstract

SecM, a bacterial secretion monitor protein, contains a specific amino acid sequence at its C-terminus, called arrest sequence, which interacts with the ribosomal tunnel and arrests its own translation. The arrest sequence is sufficient and necessary for stable translation arrest. However, some previous studies have suggested that the nascent chain outside the ribosome affects the stability of translation arrest. To clarify this issue, we performed *in vitro* translation assays with HaloTag proteins fused to the C-terminal fragment of *E*. *coli* SecM containing the arrest sequence or the full-length SecM. We showed that the translation of HaloTag proteins, which are fused to the fragment, is not effectively arrested, whereas the translation of HaloTag protein fused to full-length SecM is arrested efficiently. In addition, we observed that the nascent SecM chain outside the ribosome markedly stabilizes the translation arrest. These results indicate that changes in the nascent polypeptide chain outside the ribosome can affect the stability of translation arrest; the nascent SecM chain outside the ribosome stabilizes the translation arrest.

## Introduction

Recent studies have revealed that ribosomes do not always translate mRNAs at a constant rate. The biased codon usage and specific mRNA sequences can change the rate of polypeptide elongation [1, 2]. Sometimes the nascent chains halt the translation elongation or termination to regulate gene expression. This phenomenon, known as translation arrest, is observed in several species of both prokaryotic and eukaryotic organisms [3]. Translation arrest mediated by SecM in *Escherichia coli* has been studied most often and is best-characterized.

The *E*. *coli* SecM is a 170-amino acid (aa) secretion monitor protein. This protein regulates the translation of the downstream gene, *secA*, in response to protein secretion activity in the cell [4]. This gene encodes an ATPase that drives protein translocation. When a cell is secretion-competent, SecM evokes a transient translation arrest that is executed by physical pulling of Sec translocation apparatus [5]. Under such circumstances, the SecA ribosome-binding site is masked by the secondary structure of the mRNA, and the translation is suppressed [6, 7]. However, under secretion-limiting conditions, the SecM translation is subjected to prolonged arrest, allowing *secA* translation by altering the structure of *secM-secA* mRNA [7].

This arrest is evoked by the translation of the C-terminal specific sequence (^150^FSTPVWISQAQGIRAGP^166^) of SecM, called arrest sequence [8]. The ribosome stalls when the Pro166 codon is positioned at the A site. In other words, the stalled ribosome has the polypeptidyl-tRNA at the P site and unreacted Pro-tRNA^Pro^ at the A site of the ribosome [9, 10]. Mutations of the key residues in the arrest sequence reduce (Phe150, Trp155, Ile156, Gly161, Ile162 and Ala164) or impair (Arg163, Gly165 and Pro166) the translation arrest [8, 11, 12]. This sequence also causes translation arrest of unrelated proteins, which can be utilised to generate nascent chain-ribosome complexes [13–24]. Consequently, it is widely accepted that the SecM arrest sequence is sufficient and necessary for a sustained translation arrest.

**Fig 1:**
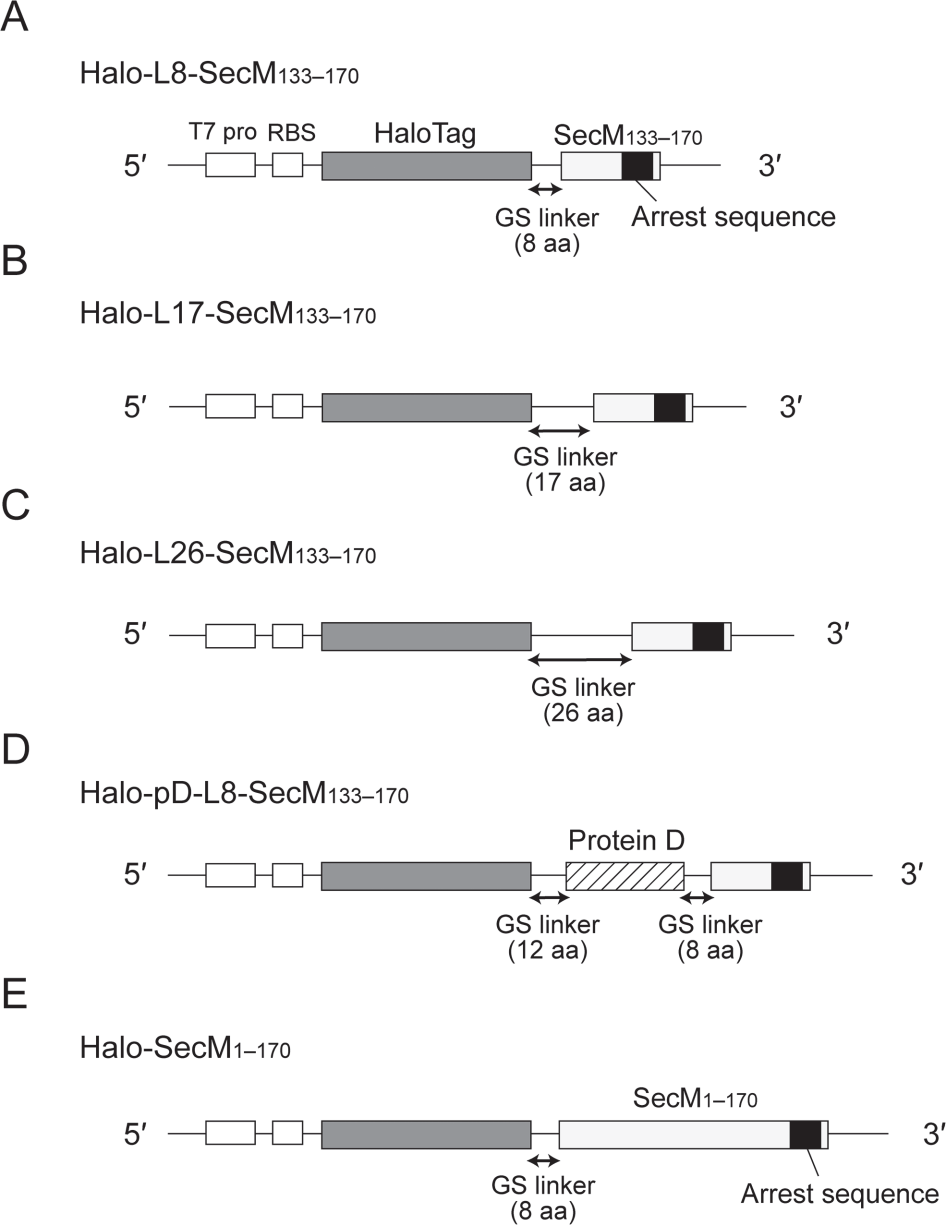
DNA constructs used for in vitro translation of HaloTag proteins harbouring the E. coli SecM arrest sequence. A T7 promoter (T7 pro) and a ribosome-binding site (RBS) are located upstream from the gene encoding HaloTag protein fused via a spacer sequence to the C-terminal sequence of *E*. *coli* SecM (residues 133–170; SecM_133–170_) or *E*. *coli* full-length SecM (residues 1–170; SecM_1–170_). (**A**) DNA construct for *in vitro* translation of Halo-L8-SecM_133–170_. The spacer sequence consists of an 8-aa glycine–serine (GS) linker (GSGGGSGS). (**B**) DNA construct for *in vitro* translation of Halo-L17-SecM_133–170_. The spacer sequence is a 17-aa GS linker (GSGGGSGGGSGGGSGGS). (**C**) DNA construct for *in vitro* translation of Halo-L26-SecM_133–170_. The spacer sequence consists of a 26-aa GS linker (GSGGGSGGGSGGGSGGGSGGGSGGGS). (**D**) DNA construct for *in vitro* translation of Halo-pD-L8-SecM_133–170_. The spacer sequence is composed of a 12-aa GS linker (GSGGGSGGGSMG), a monomeric version of protein D from the bacteriophage λ (residues 21–110) and an 8-aa GS linker (GSGGGSGS) [17, 20, 28]. (**E**) DNA construct for *in vitro* translation of Halo-SecM_1–170_. SecM_1–170_ is fused to the C-terminus of HaloTag via an 8-aa GS linker (GSGGGSGS). The molecular masses of Halo-L8-SecM_133–170_, Halo-L17-SecM_133–170_, Halo-L26-SecM_133–170_, Halo-pD-L8-SecM_133–170_ and Halo-SecM_1–170_, calculated from the deduced amino acid sequences, were 38, 39, 40, 49 and 53 kDa, respectively.

However, some of the existing data suggest that the arrest sequence alone is not enough to provide a stable translation arrest [21, 22]. Evans *et al*. have shown that the efficiency of translation arrest changes depending on the protein displayed on the ribosome [13]. We hypothesized that the nascent chain outside the ribosome affects the efficiency of translation arrest. To test this hypothesis, we performed *in vitro* translation assays using HaloTag proteins fused to either the *E*. *coli* SecM C-terminal sequence containing the arrest sequence or full-length SecM (Fig. 1). As a result, we found that changes in the nascent polypeptide chain outside the ribosome can affect the stability of translation arrest and the nascent SecM chain outside the ribosome helps to stabilize the arrest.

## Materials and Methods

### Reagents

PrimeSTAR HS DNA Polymerase was obtained from Takara Bio, Inc. The PURExpress ΔRibosome Kit, nuclease-free water and HaloTag TMR Ligand were purchased from New England Biolabs, QIAGEN and Promega, respectively. SUPERase-In RNase Inhibitor, NuPAGE 10% Bis-Tris Gel, NuPAGE MOPS SDS Running Buffer and RNA*secure* were purchased from Life Technologies. Molecular weight markers (Precision Plus Protein Prestained Standards) and puromycin were purchased from Bio-Rad and Sigma-Aldrich, respectively. Anti-mouse IgG conjugated with HiLyte Fluor 555 [Anti-IgG (H + L), Mouse, Rabbit-Poly, HiLyte Fluor 555] was obtained from AnaSpec, Inc. Other reagents were purchased from Wako Pure Chemicals Industries, Ltd.

### Preparation of DNA templates for *in vitro* translation

The T7-based expression plasmids for HaloTag proteins harbouring the *E*. *coli* SecM arrest sequence were constructed as described in S1 Document. Primer sequences used in this study are listed in S1 Table. The plasmids were used as templates for PCR amplification with primer 1, 5′-GAAATTAATACGACTCACTATAGGGG-3′ and primer 2, 5′-GCTAGTTATTGC TCAGCGG-3′. Primer 1 contained the T7 promoter sequence (underlined) and primer 2 overlapped the T7 terminator sequence. The PCR products were used as templates for *in vitro* translation of the proteins [25].

### *In vitro* translation

**Fig 2:**
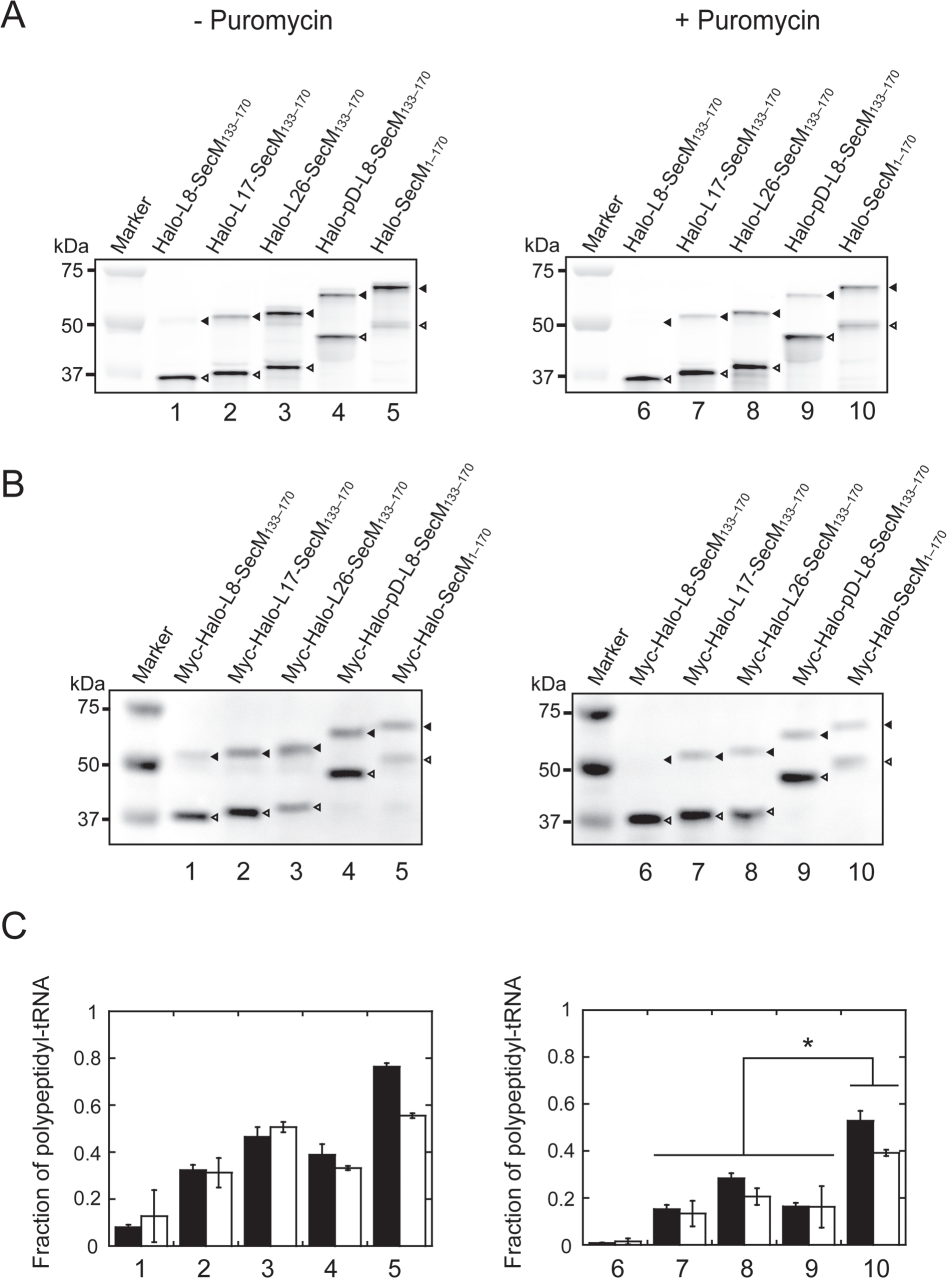
In vitro translation of HaloTag proteins harbouring the arrest sequence. (**A**) Halo-L8-SecM_133–170_ (lane 1), Halo-L17-SecM_133–170_ (lane 2), Halo-L26-SecM_133–170_ (lane 3), Halo-pD-L8-SecM_133–170_ (lane 4) and Halo-SecM_1–170_ (lane 5) were translated in the presence of HaloTag TMR Ligand using the PURExpress ΔRibosome Kit at 37°C for 20 min. Puromycin (1 mg/mL) was added to the reaction mixture at 0 min, and the reaction mixture was incubated at 37°C for 3 min. Aliquots were withdrawn before the addition of puromycin and after 3-min incubation and subjected to NuPAGE. Polypeptides labelled with HaloTag TMR Ligand were detected using Molecular Imager FX. Black and white arrowheads indicate the translation arrest products (polypeptidyl-tRNA) and released products, respectively. The results shown are representative of three independent experiments with similar results. (**B**) Myc-Halo-L8-SecM_133–170_ (lane 1), myc-Halo-L17-SecM_133–170_ (lane 2), myc-Halo-L26-SecM_133–170_ (lane 3), myc-Halo-pD-L8-SecM_133–170_ (lane 4) and myc-Halo-SecM_1–170_ (lane 5) were translated in the absence of HaloTag TMR Ligand using the PURExpress ΔRibosome Kit at 37°C for 20 min. Puromycin (1 mg/mL) was added at 0 min, and the reaction mixture was incubated at 37°C for 3 min. Aliquots were withdrawn before the addition of puromycin and after a 3-min incubation and subjected to NuPAGE. Myc-tagged polypeptides were detected by western blotting with anti-c-myc-tag. Black and white arrowheads indicate the translation arrest products (polypeptidyl-tRNA) and released products, respectively. The results shown are representative of three independent experiments with similar results. (**C**) Fractions of translation arrest products in the absence (left) and the presence of puromycin (right). Filled bars, fluorescence detection using HaloTag TMR Ligand; open bars, detection by western blotting. Error bars represent the standard deviation (SD) of three independent experiments. The asterisk indicates statistical significance as determined by the Student's *t*-test (*p* < 0.05).

**Fig 3:**
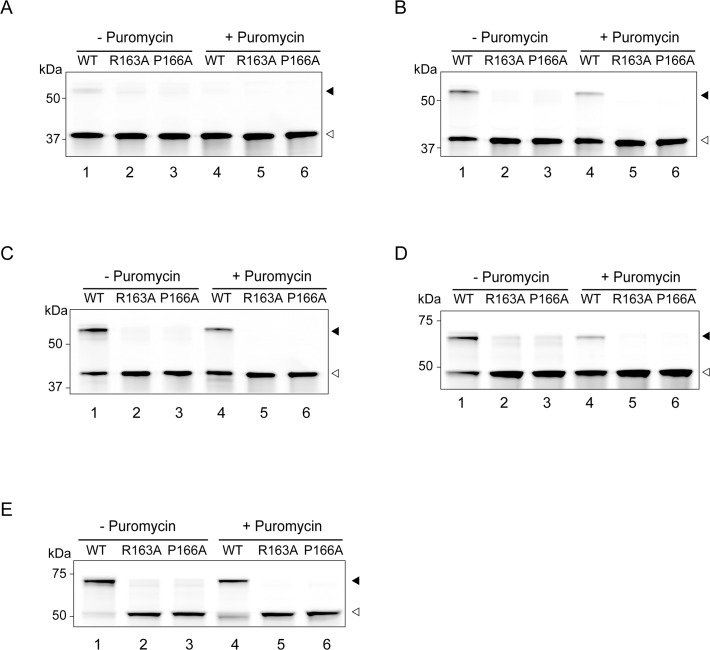
In vitro translation of HaloTag proteins with mutated arrest sequence. Each protein construct, with or without a mutation (R163A or P166A) in the arrest sequence, was translated in the presence of HaloTag TMR Ligand using the PURExpress ΔRibosome Kit at 37°C for 20 min. Puromycin (1 mg/mL) was added at 0 min, and the reaction mixture incubated at 37°C for 3 min. Aliquots were withdrawn before and 3 min after the addition of puromycin and subjected to NuPAGE. Polypeptides labelled with HaloTag TMR Ligand were detected using Molecular Imager FX. **A**, Halo-L8-SecM_133–170_; **B**, Halo-L17-SecM_133–170_; **C**, Halo-L26-SecM_133–170_; **D**, Halo-pD-L8-SecM_133–170_; **E**, Halo-SecM_1–170_. Black and white arrowheads indicate the translation arrest products (polypeptidyl-tRNA) and released products, respectively. The results shown are representative of three independent experiments with similar results.

**Fig 4:**
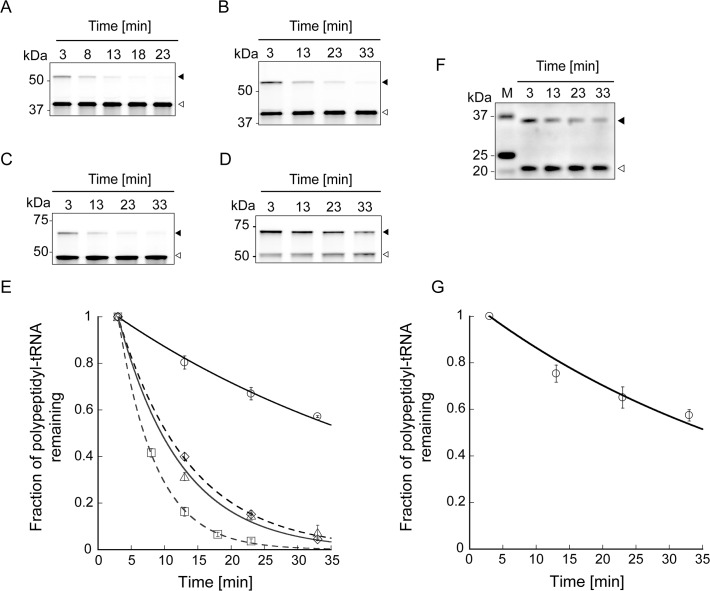
Lifetimes of the translation arrest of HaloTag proteins harbouring the arrest sequence. (**A**-**D**) Time-course analyses of polypeptidyl-tRNA remaining after the addition of puromycin. Halo-L17-SecM_133–170_ (**A**), Halo-L26-SecM_133–170_ (**B**), Halo-pD-L8-SecM_133–170_ (**C**) and Halo-SecM_1–170_ (**D**) were translated in the presence of HaloTag TMR Ligand using the PURExpress ΔRibosome Kit at 37°C for 20 min. Puromycin (1 mg/mL) was added to the reaction mixture at 0 min, and the mixture was incubated at 37°C. Aliquots removed at the indicated time points were subjected to NuPAGE. Polypeptides labelled with HaloTag TMR Ligand were detected using Molecular Imager FX. Black and white arrowheads indicate the translation arrest products (polypeptidyl-tRNA) and released products, respectively. (**E**) Plots of the fraction of polypeptidyl-tRNA remaining in the presence of puromycin as a function of time. Squares, Halo-L17-SecM_133–170_; diamonds, Halo-L26-SecM_133–170_; triangles, Halo-pD-L8-SecM_133–170_; circles, Halo-SecM_1–170_. Data points represent means ± SD of three independent experiments. The solid and dotted lines show the fit to the data obtained using a single exponential function. The lifetimes of the translation arrest of Halo-L17-SecM_133–170_, Halo-L26-SecM_133–170_, Halo-pD-L8-SecM_133–170_ and Halo-SecM_1–170_ were 5.6 ± 0.066, 11 ± 0.22, 9.4 ± 0.63 and 51 ± 1.6 min, respectively (the errors represent fitting errors). (**F**) Time-course analysis of myc-SecM_1–170_ polypeptidyl-tRNA remaining after the addition of puromycin. Myc-SecM_1–170_ was translated using the PURExpress ΔRibosome Kit at 37°C for 40 min. Puromycin (1 mg/mL) was added at 0 min, and the mixture was incubated at 37°C. Aliquots were withdrawn at indicated time points and subjected to NuPAGE. Myc-SecM_1–170_ was detected by western blotting with anti-c-myc-tag. Black and white arrowheads indicate the translation arrest products (polypeptidyl-tRNA) and released products, respectively. (**G**) The fraction of myc-SecM_1–170_ polypeptidyl-tRNA remaining in the presence of puromycin as a function of time. Data points with error bars represent means ± SD for three independent experiments. The solid line shows the fit to the data obtained using a single exponential function. The lifetime of the translation arrest of myc-SecM_1–170_ was 48 min ± 4.3 min (the error corresponds to fitting error).

HaloTag proteins harbouring the SecM arrest sequence were synthesized using the PURExpress ΔRibosome Kit. First, the reaction mixtures without ribosomes were assembled. The mixture for reaction with HaloTag TMR Ligand contained 4.0 μL of Solution A, 1.2 μL of Factor Mix, 2.0 μL of template DNA, 0.5 μL of 20 μM HaloTag TMR Ligand, 0.5 μL of 20 U/μL RNase inhibitor and 1.3 μL of nuclease-free water in a 10 μL reaction. The mixture for reaction without HaloTag TMR Ligand contained 4.0 μL of Solution A, 1.2 μL of Factor Mix, 2.0 μL of template DNA, 0.5 μL of 20 U/μL RNase inhibitor and 1.8 μL of nuclease-free water in a 10 μL reaction. Then, the mixtures were incubated at 37°C for 10 min to allow transcription. After the incubation, 0.5 μL of 13.3 μM ribosomes was added to the mixture, and the mixture was incubated at 37°C for 20 or 40 min to allow translation. Subsequently, puromycin was added to a final concentration of 1 mg/mL, and the mixture was incubated at 37°C. For the experiments described in Figs. 2 and 3, aliquots were withdrawn from the mixture before and 3 min after the addition of puromycin. For the experiments illustrated in Fig. 4, aliquots were withdrawn from the mixture at the indicated times after the addition of puromycin.

### SDS-PAGE analysis

To detect polypeptidyl-tRNA, translation products were subjected to SDS-PAGE at a neutral pH (NuPAGE) [9]. The Laemmli sample buffer (62.5 mM Tris-HCl, pH6.8; 2% SDS; 10% glycerol and 5% β-mercaptoethanol) was treated with RNA*secure* for 10 min at 60°C to inactivate contaminating RNase [26]. The translation products were denatured with the buffer without heat treatment [26] and separated on a NuPAGE 10% Bis-Tris gel in MOPS running buffer at 4°C. Polypeptides labelled with HaloTag TMR Ligand were visualized using Molecular Imager FX (Bio-Rad). Myc-tagged polypeptides were detected by western blotting using anti-c-myc antibody (9E10) and anti-mouse IgG conjugated with HiLyte Fluor 555. Band intensities were measured using ImageJ (http://imagej.nih.gov/ij/). The fraction of the translation arrest product (*f*) was calculated using the following Equation 1:

$$f\;=\;\frac{I_{a}}{I_{a}+I_{f}}$$ (1),

where *I*_a_ and *I*_r_ are the band intensities of the translation arrest product and released product, respectively.

### Lifetime analysis of translation arrest

The lifetimes of translation arrest of HaloTag proteins harbouring the SecM arrest sequence were determined by fitting a single exponential curve to the plot of the fraction of polypeptidyl-tRNA remaining in the presence of puromycin against incubation time. Data fitting was performed using the KaleidaGraph program (Synergy Software).

## Results and Discussion

We found that translation of SecM C-terminal peptide was arrested in a similar manner to that of a native SecM (S1 Fig.), indicating that the arrest sequence is necessary and sufficient for translation arrest, which is consistent with previous studies [8, 27, 28]. However, some of the existing data suggest that the translation of proteins fused to the arrest sequence is not stably arrested [13, 21, 22]. We hypothesized that the nascent chain outside the ribosome affects the efficiency of translation arrest.

### DNA constructs for *in vitro* translation of HaloTag proteins harbouring the *E*. *coli* SecM arrest sequence

To examine the effect of the nascent chain outside the ribosome on SecM-mediated translation arrest, we prepared DNA constructs for *in vitro* transcription and translation of HaloTag harbouring the *E*. *coli* SecM arrest sequence (Fig. 1). The constructs contained a T7 promoter and ribosome-binding site upstream of the gene encoding HaloTag protein. The HaloTag sequence was fused to either the C-terminal sequence of SecM (residues 133–170; SecM_133–170_) or full-length SecM (residues 1–170; SecM_1–170_), via a spacer sequence. HaloTag is a 34-kDa monomeric protein derived from a bacterial haloalkane dehalogenase, which was engineered to form efficiently a covalent bond with the HaloTag ligand [29]. SecM_133–170_, which contains the arrest sequence (residues 150–166), is probably within the ribosomal exit tunnel upon translation arrest [11]. The spacer sequence provides sufficient distance between the HaloTag protein and ribosome to allow the correct protein folding [19, 30]. Halo-L8-SecM_133–170_, Halo-L17-SecM_133–170_ and Halo-L26-SecM_133–170_ have spacers comprising 8-, 17- and 26-aa glycine–serine (GS) linker, respectively (Fig. 1A, B and C). Halo-pD-L8-SecM_133–170_ has a spacer comrpising a 12-aa GS linker, a monomeric version of protein D from the bacteriophage λ (residues 21–110; pD) and an 8-aa GS linker [17, 20, 30] (Fig. 1D). Halo-SecM_1–170_ has an 8-aa GS spacer (Fig. 1E). The molecular masses of Halo-L8-SecM_133–170_, Halo-L17-SecM_133–170_, Halo-L26-SecM_133–170_, Halo-pD-L8-SecM_133–170_ and Halo-SecM_1–170_, which were calculated from the deduced aa sequence, were 38, 39, 40, 49 and 53 kDa, respectively. The constructs with an N-terminal myc-tag were also prepared.

### *In vitro* translation of HaloTag proteins harbouring the *E*. *coli* SecM arrest sequence

The DNA constructs were transcribed and translated with or without HaloTag TMR Ligand using a reconstituted *in vitro* transcription–translation system from *E*. *coli* [31]. HaloTag is a protein with improved folding and solubility [32], which rapidly reacts with its ligand (*k*_on_ = 2 × 10^7^ M^−1^ s^−1^) [29]. If nascent HaloTag is correctly folded outside the ribosome exit tunnel, it will be covalently labelled with its fluorescent ligand within 1 s. The translation products with or without translation inhibitor, puromycin (a structural analogue of aminoacyl-tRNA) were subjected to SDS-PAGE at a neutral pH (NuPAGE) to detect the product of translation arrest, polypeptidyl-tRNA [9, 26] (Fig. 2A and 2B). Polypeptides were visualized using HaloTag TMR Ligand or western blotting with anti-c-myc-tag antibody to detect the incorrectly folded products. The fractions of the translation arrest products obtained with or without puromycin are shown in Fig. 2C.

Translation of Halo-L8-SecM_133–170_ produced one faint and one strong band with apparent molecular masses of approximately 53 and 38 kDa, corresponding to the arrested polypeptidyl-tRNA and the polypeptides released from the ribosome, respectively (Fig. 2A and 2C, lane 1). The arrested polypeptidyl-tRNA is not effectively released from the ribosome after the addition of puromycin because Pro-tRNA^Pro^ occupies the A site of the ribosome and blocks the access of puromycin to this site [9–11]. However, the arrested polypeptidyl-tRNA disappeared almost completely within 3 min after the addition of puromycin (Fig. 2A and 2C, lane 6). The same results were obtained by western blotting for Halo-L8-SecM_133–170_ with an N-terminal myc-tag (Fig. 2B and 2C, lane 1 and 6). These results indicate that HaloTag-L8-SecM_133–170_ has little arrest potential.

Translation of Halo-L17-SecM_133–170_, Halo-L26-SecM_133–170_ and Halo-pD-L8-SecM_133–170_ produced two bands: the upper band derived from the arrested polypeptidyl-tRNA and the lower, the polypeptide released from the ribosome (Fig. 2A and 2C, lane 2–4). In each construct, approximately half of the upper band survived the treatment with puromycin (Fig. 2A and 2C, lane 2–4 and 7–9). Comparable results were obtained by western blotting using the myc-tagged constructs (Fig. 2B and 2C, lane 2–4 and 7–9). These results suggest that the Halo-L17-SecM_133–170_, Halo-L26-SecM_133–170_ and Halo-pD-L8-SecM_133–170_ have a weak arrest potential and that their translation can be arrested in a manner similar to that of a native SecM.

Translation of Halo-SecM_1–170_ yielded strong and weak bands corresponding to the arrested polypeptidyl-tRNA and polypeptide released from the ribosome, respectively (Fig. 2A and 2C, lane 5). Most of the arrested polypeptidyl-tRNA was resistant to puromycin (Fig. 2A and 2C, lane 5 and 10), showing that Halo-SecM_1–170_ is more likely to be translated as the arrested form than other constructs. Similar results were obtained using western blotting (Fig. 2B and 2C, lane 5 and 10).

### *In vitro* translation of HaloTag proteins with mutated arrest sequence

Similar experiments were performed using the constructs with R163A or P166A mutation in the arrest sequence (Fig. 3). Previous studies have shown that R163A and P166A mutations completely abolish SecM-mediated translation arrest [8, 11, 12]. Introduction of R163A and P166A mutations into the constructs eliminated the bands derived from the arrested polypeptidyl-tRNA (Fig. 3). These results indicate that the translation of Halo-L17-SecM_133–170_, Halo-L26-SecM_133–170_, Halo-pD-L8-SecM_133–170_ and Halo-SecM_1–170_ is arrested in an arrest sequence-dependent manner. These data, in conjunction with the data shown in Figs. 2 and 3, demonstrate that the efficiency of translation arrest can be affected by the nascent chain outside the ribosome in an arrest sequence-dependent manner.

### Lifetime of the translation arrest of HaloTag proteins harbouring the arrest sequence

Next, to evaluate the stability of the translation arrest of Halo-L17-SecM_133–170_, Halo-L26-SecM_133–170_, Halo-pD-L8-SecM_133–170_ and Halo-SecM_1–170_, the lifetimes of the translation arrest were examined (Fig. 4). The translation products were treated with 1 mg/mL puromycin at 37°C to inhibit translation. Aliquots of the mixture, obtained at the indicated time points, were subjected to NuPAGE (Fig. 4A–D). When the translation arrest is released and the peptide elongation resumes, puromycin can enter the A site of the ribosome, producing polypeptidyl-puromycin. Therefore, the arrested polypeptidyl-tRNA is converted to the polypeptide and released from the ribosome as the incubation progresses. The lifetime of each translation arrest was determined by fitting a single exponential curve to the plot of the fraction of polypeptidyl-tRNA remaining in the presence of puromycin against incubation time with puromycin (Fig. 4E). The results were as follows: 5.6 ± 0.066 min for Halo-L17-SecM_133–170_, 11 ± 0.22 min for Halo-L26-SecM_133–170_, 9.4 ± 0.63 min for Halo-pD-L8-SecM_133–170_ and 51 ± 1.6 min for Halo-SecM_1–170_ (the errors represent fitting errors). The lifetimes of Halo-SecM_1–170_ and N-terminal myc-tagged SecM (48 ± 4.3 min) were similar (Fig. 4G), indicating that their translation is arrested in the same manner. We concluded that the changes in the nascent chain outside the ribosome affects the stability of translation arrest and the nascent SecM chain outside the ribosome stabilizes the translation arrest.

### The effect of the nascent chain outside the ribosome on the translation arrest

We showed that the changes in the nascent chain outside the ribosome affects the stability of translation arrest and the nascent SecM chain helps to stabilize the translation arrest (Figs. 2–4). We can assume that this nascent chain stabilizes the translation arrest by interacting with the ribosome. The majority of the annotated SecM proteins are basic proteins; the *E*. *coli* SecM also shares this inherent property (pI = 9.98). Because the ribosome has a highly negatively charged surface, it is likely that the nascent SecM chain associates directly with the ribosome via electrostatic interactions, resulting in a sustained translation arrest. This translation arrest induces the expression of the *secA* gene, which is downstream of *secM* [7]. Then, the SecA proteins in association with SecYEG translocon disrupt the interaction between SecM and ribosome in the Sec-mediated export process [7, 8]. The residues 100–109 in SecM might work as ‘release mediator’ to alleviate the arrest [33]. We can conclude that the nascent SecM chain outside the ribosome plays an important role in the regulation of the translation arrest.

We also observed that the efficiency and stability of translation arrest correlate with the length of the spacer sequence between HaloTag protein and SecM C-terminal sequence (Figs. 2 and 4). In barnase-ubiquitin fusion proteins, the conformational strain can be spread over the entire polypeptide when the protein folds [34, 35]. Thus, it is likely that upon translation of Halo-L8-SecM_133–170_, Halo-L17-SecM_133–170_, Halo-L26-SecM_133–170_ or Halo-pD-L8-SecM_133–170_, the nascent SecM_133–170_ is pulled by the extension of the GS linker when HaloTag and pD fold cotranslationally. The pulling force might decrease with the increasing linker length [34]. Woolhead *et al*. [11] have reported that the C-terminal sequence of SecM adopts a compact conformation in the ribosome exit tunnel, which is essential for the translation arrest. The pulling force exerted on the nascent chain would interfere with the conformational transition of the SecM C-terminal sequence. It has been suggested that the SecM arrest sequence is susceptible to a pulling force exerted by the nascent chain outside the ribosome [22, 24]. In the light of these reports and our own findings, SecM appears to be involved in a remarkably sophisticated system regulating its translation.

## Supporting Information

S1 Document Supporting Materials and Methods. (DOCX)

S1 Fig In vitro translation of SecM and the C-terminal sequence. (A) Wild-type and mutant SecM proteins were translated in the presence of [^35^S]-methionine (0.31 μCi/μL) using the PURExpress ΔRibosome Kit at 37°C for 20 min. Puromycin (1 mg/mL) was added to the reaction mixture at 0 min and the mixture was incubated at 37°C for 3 min. Aliquots were withdrawn before the addition of puromycin and after 3-min incubation and separated on a NuPAGE 12% Bis-Tris gel in MES running buffer at 4°C. Polypeptides were detected by autoradiography. (B) The C-terminal peptide of SecM (residues 133–170; SecM_133–170_), with or without a mutation (R163A or P166A) in the arrest sequence, was translated in the presence of [^35^S]-methionine (0.31 μCi/μL), using the PURExpress ΔRibosome Kit at 37°C for 20 min. Puromycin (1 mg/mL) was added to the reaction mixture at 0 min and the reaction mixture was incubated at 37°C for 3 min. Aliquots were withdrawn before the addition of puromycin and after 3-min incubation and separated on a NuPAGE 12% Bis-Tris gel in MES running buffer at 4°C. Polypeptides were detected by autoradiography. Black and white arrowheads indicate the translation arrest products (polypeptidyl-tRNA) and released products, respectively. Single asterisks indicate bands corresponding to the methionine-charged tRNA and a double asterisk indicates bands corresponding to a translation by-product (probably peptidyl-tRNA).
(TIF)

S1 Table Primer sequences used in this study. Restriction enzyme recognition sites are underlined. The bold sequences encode myc-tag. The mutated codons are indicated as boxed nucleotides.
(DOCX)
