# Supplementary material for: Nascent SecM Chain Outside the Ribosome Reinforces Translation Arrest
Source: PLoS One. 2015 Mar 25;10(3):e0122017. doi: 10.1371/journal.pone.0122017 (PMC4373844; doi:10.1371/journal.pone.0122017)
Supplement: S1 Document — (DOCX) [file pone.0122017.s001.docx]

**S1 File. Supporting Materials and Methods**

**Cloning of *E. coli* *secM* gene**

The gene encoding SecM was amplified from *E. coli* genomic DNA using SecM_F and SecM_R primers to introduce a *Hin*d III restriction site at the 5′-end and a *Bam*H I restriction site at the 3′-end [1] (Table S1). The amplified fragment was digested with *Hin*d III and *Bam*H I and then ligated to the same sites in the pTA2 vector (TOYOBO) to yield pTA-SecM.

**Construction of expression plasmid for Halo-L8-SecM_133–170_**

The gene encoding HaloTag was amplified from pFN18A HaloTag T7 Flexi Vector (Promega) using HaloTag_F and HaloTag_R primers. The gene encoding SecM_133–170_ was amplified from pTA-SecM using SecM_F2 and SecM_R2 primers (Table S1). The amplified fragments were used as templates with HaloTag_F and SecM_R2 primers to amplify the entire Halo-L8-SecM_133–170_ gene (Table S1). The amplified fragment was digested with the *Nde* I and *Hin*d III and ligated to the same sites in the pET21c vector (Novagen).

R163A and P166A mutants of Halo-L8-SecM_133–170_ were obtained using the QuikChange site-directed mutagenesis kit (Agilent Technologies). Primers used for mutagenesis are listed in Table S1.

**Construction of expression plasmid for Halo-L17-SecM_133–170_**

The gene encoding HaloTag was amplified from pFN18A HaloTag T7 Flexi Vector using HaloTag_F and HaloTag_R2 primers. The gene encoding GS linker (17 aa) and SecM_133–170_ was amplified from the synthetic gene cloned into the vector (Integrated DNA Technologies) using Linker_F and SecM_R2 primers (Table S1). The amplified fragments were used as templates with HaloTag_F and SecM_R2 primers to amplify the entire Halo-L17-SecM_133–170_ gene (Table S1). The amplified fragment was digested with the *Nde* I and *Hin*d III and ligated to the same sites in the pET23a vector (Novagen).

R163A and P166A mutants of Halo-L17-SecM_133–170_ were obtained using the QuikChange site-directed mutagenesis kit. Primers used for mutagenesis are listed in Table S1.

**Construction of expression plasmid for Halo-L26-SecM_133–170_**

The gene encoding HaloTag was amplified from pFN18A HaloTag T7 Flexi Vector using HaloTag_F and HaloTag_R2 primers. The gene encoding GS linker (26 aa) and SecM_133–170_ was amplified from the synthetic gene cloned into the vector (Integrated DNA Technologies) using Linker_F and SecM_R2 (Table S1). The amplified fragments were used as templates with HaloTag_F and SecM_R2 primers to amplify the entire Halo-L26-SecM_133–170_ gene (Table S1). The amplified fragment was digested with the *Nde* I and *Hin*d III and ligated to the same sites in the pET21c vector.

R163A and P166A mutants of Halo-L26-SecM_133–170_ were obtained using the QuikChange site-directed mutagenesis kit. Primers used for mutagenesis are listed in Table S1.

**Construction of expression plasmid for Halo-pD-L8-SecM_133–170_**

The gene encoding HaloTag was amplified from pFN18A HaloTag T7 Flexi Vector using HaloTag_F and HaloTag_R primers to introduce an *Nde* I restriction site at the 5′-end and a *Bam*H I restriction site at the 3′-end (Table S1). The amplified fragment was digested with *Nde* I and *Bam*H I and then introduced into the same sites in the expression plasmid for GFPuv3-pD-SecM_148–166_ [2, 3]. The resultant plasmid was designated pHalo-pD-SecM_148–166_.

The gene encoding HaloTag and pD was amplified from pHalo-pD-SecM_148–166_ using HaloTag_F and pD_R primers. The gene encoding SecM_133–170_ was amplified from pTA-SecM using SecM_F2 and SecM_R2 primers (Table S1). The amplified fragments were used as templates with HaloTag_F and SecM_R2 primers to amplify the entire Halo-pD-L8-SecM_133–170_ gene (Table S1). The amplified fragment was digested with the *Nde* I and *Hin*d III and ligated to the same sites in the pET23a vector.

R163A and P166A mutants of Halo-pD-L8-SecM_133–170_ were obtained using the QuikChange site-directed mutagenesis kit. Primers used for mutagenesis are listed in Table S1.

**Construction of expression plasmid for Halo-SecM_1–170_**

The gene encoding HaloTag protein was amplified from pFN18A HaloTag T7 Flexi Vector using HaloTag_F and HaloTag_R to introduce an *Nde* I restriction site at the 5′-end and a *Bam*H I restriction site at the 3′-end (Table S1). The gene encoding SecM_1–170_ was amplified from pTA-SecM using SecM_F3 and SecM_R2 primers to introduce a *Bam*H I restriction site at the 5′-end and a *Hin*d III restriction site at the 3′-end (Table S1). The amplified fragments were digested with the appropriate restriction enzymes and co-ligated to the *Nde* I and *Hin*d III restriction sites in the pET21c vector.

R163A and P166A mutants of Halo-SecM_1–170_ were obtained using the QuikChange site-directed mutagenesis kit. Primers used for mutagenesis are listed in Table S1.

**Construction of expression plasmid for myc-tagged proteins**

The genes encoding Halo-L8-SecM_133–170_, Halo-L17-SecM_133–170_, Halo-L26-SecM_133–170_, Halo-pD-L8-SecM_133–170_ and Halo-SecM_1–170_ were amplified from the expression plasmids using HaloTag_F2 and SecM_F2 primers to introduce an *Nde* I restriction site at the 5′-end and a *Hin*d III restriction site at the 3′-end (Table S1). The amplified fragments were digested with *Nde* I and *Hin*d III and then introduced into the same sites in the pET21c or pET23a vector.

The gene encoding SecM_1–170_ was amplified from pTA-SecM using SecM_F4 and SecM_R3 primers to introduce an *Nde* I restriction site at the 5′-end and a *Bam*H I restriction site at the 3′-end (Table S1). The amplified gene was digested with *Nde* I and *Bam*H I and then ligated to the same sites in the pET23a vector.

**Construction of expression plasmids for SecM and SecM**_133–170_

The expression plasmids for SecM and SecM_133-170_ were obtained using the KOD -Plus- Mutagenesis Kit (Toyobo), with the expression plasmid for myc-SecM as a template. The primer sets used (Δmyc_#1 and Δmyc_#2/133-170_#2) are listed in Table S1. In SecM_133–170_, two consecutive methionines were fused next to the start methionine to increase the labelling efficiency.

**References**

1. Nakatogawa H, Ito K (2001) Secretion monitor, SecM, undergoes self-translation arrest in the cytosol. Mol Cell 7: 185−192.
2. Uemura S, Iizuka R, Ueno T, Shimizu Y, Taguchi H, Ueda T, Puglisi JD, Funatsu T (2008) Single-molecule imaging of full protein synthesis by immobilized ribosomes. Nucleic Acids Res 36: e70.
3. Iizuka, R, Funatsu T, Uemura S (2010) Real-time single-molecule observation of green fluorescent protein synthesis by immobilized ribosomes. Methods Mol Biol 778: 215−228.
