# Supplementary figures and images for: Nascent SecM Chain Outside the Ribosome Reinforces Translation Arrest
Source: PLoS One. 2015 Mar 25;10(3):e0122017. doi: 10.1371/journal.pone.0122017 (PMC4373844; doi:10.1371/journal.pone.0122017)

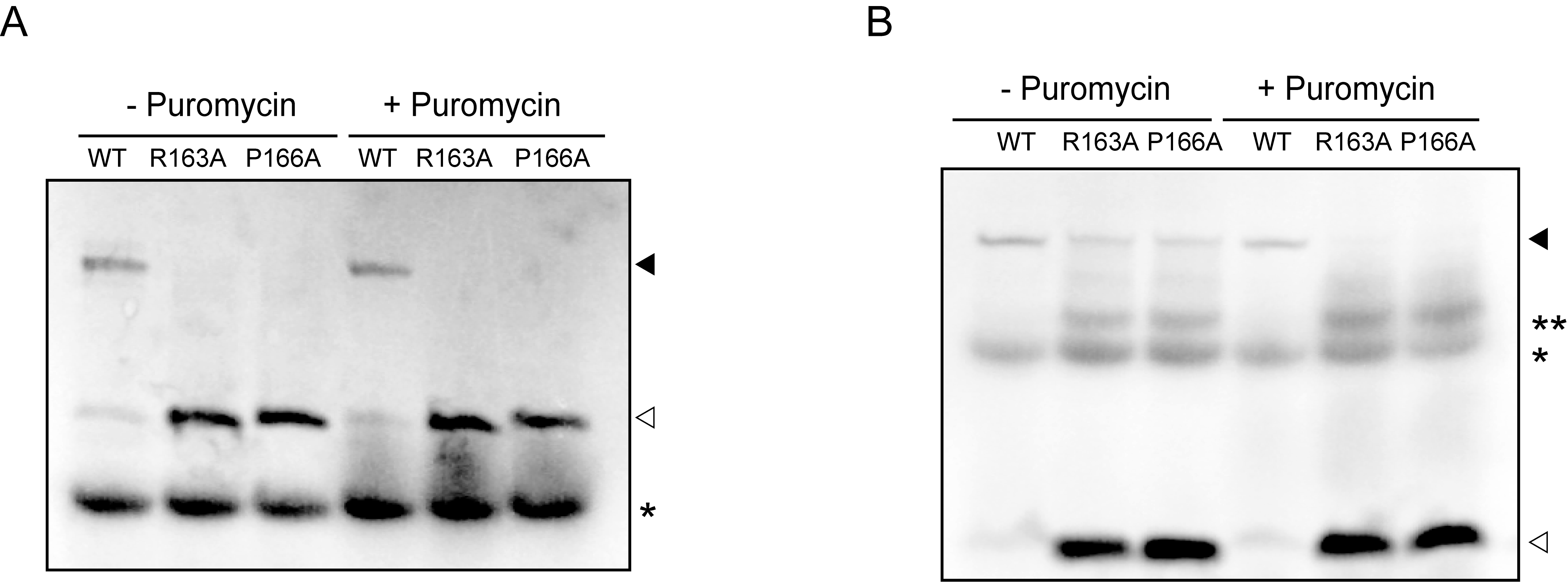

Supplement: S1 Fig — (A) Wild-type and mutant SecM proteins were translated in the presence of [35S]-methionine (0.31 μCi/μL) using the PURExpress ΔRibosome Kit at 37°C for 20 min. Puromycin (1 mg/mL) was added to the reaction mixture at 0 min and the mixture was incubated at 37°C for 3 min. Aliquots were withdrawn before the addition of puromycin and after 3-min incubation and separated on a NuPAGE 12% Bis-Tris gel in MES running buffer at 4°C. Polypeptides were detected by autoradiography. (B) The C-terminal peptide of SecM (residues 133–170; SecM133–170), with or without a mutation (R163A or P166A) in the arrest sequence, was translated in the presence of [35S]-methionine (0.31 μCi/μL), using the PURExpress ΔRibosome Kit at 37°C for 20 min. Puromycin (1 mg/mL) was added to the reaction mixture at 0 min and the reaction mixture was incubated at 37°C for 3 min. Aliquots were withdrawn before the addition of puromycin and after 3-min incubation and separated on a NuPAGE 12% Bis-Tris gel in MES running buffer at 4°C. Polypeptides were detected by autoradiography. Black and white arrowheads indicate the translation arrest products (polypeptidyl-tRNA) and released products, respectively. Single asterisks indicate bands corresponding to the methionine-charged tRNA and a double asterisk indicates bands corresponding to a translation by-product (probably peptidyl-tRNA). (TIF) [file pone.0122017.s002.tif]
