# Supplementary material for: Nascent SecM Chain Outside the Ribosome Reinforces Translation Arrest
Source: PLoS One. 2015 Mar 25;10(3):e0122017. doi: 10.1371/journal.pone.0122017 (PMC4373844; doi:10.1371/journal.pone.0122017)
Supplement: S1 Table — Restriction enzyme recognition sites are underlined. The bold sequences encode myc-tag. The mutated codons are indicated as boxed nucleotides. (DOCX) [file pone.0122017.s003.docx]

**S1 Table. Primer sequences used in this study.**

| **Primer name** | **Sequence** | **Enzyme** |
| --- | --- | --- |
| SecM_F | 5′-CCCAAGCTTGGCAATAACGTGAGTGG-3′ | *Hin*d III |
| SecM_R | 5′-CGGGATCCATAATAAAATCTCAAACG-3′ | *Bam*H I |
| HaloTag_F | 5′-GGGAATTCCATATGGCAGAAATCGGTACTGG-3′ | *Nde* I |
| HaloTag_R | 5′-GCCAGAGCCACCGCCggatccGCCGGAAATCTCGAGCGTCG-3′ | *Bam*H I |
| SecM_F2 | 5′-GGATCCGGCGGTGGCTCTGGCTCTGAAAAGGGTTATCGCATTG-3′ | *Bam*H I |
| SecM_R2 | 5′-CCCaagcTTAGGTGAGGCGTTGAGGG-3′ | *Hin*d III |
| HaloTag_R2 | 5′-GCCGGAAATCTCGAGCGTCGACAGCC-3′ | - |
| Linker_F | 5′- CGCTGGCTGTCGACGCTCG-3′ | - |
| pD_R | 5′-GCCAGAGCCACCGCCGGATCCaacgatgctgattgccgttcc-3′ | *Bam*H I |
| SecM_F3 | 5′-CGggatccggcggtggctctggctctGTGAGTGGAATACTGACGCG-3′ | *Bam*H I |
| HaloTag_F2 | 5′-GGAATTCCATATG**GAGCAGAAACTCATCTCTGAAGAGGATCTG**GCAGAAATCGGTACTGGCTTTC-3′ | *Nde* I |
| SecM_F4 | 5′-GGAATTCCATATG**GAGCAGAAACTCATCTCTGAAGAGGATCTG**GTGAGTGGAATACTGACGCG-3′ | *Nde* I |
| SecM_R3 | 5′-CGGGATCCTTAGGTGAGGCGTTGAGGG-3′ | *Bam*H I |
| R163A_#1 | 5′-AGGCGCAAGGCATCgcTGCTGGCCCTCAAC-3′ | - |
| R163A_#2 | 5′-TTGAGGGCCAGCAgcGATGCCTTGCGCCTG-3′ | - |
| P166A_#1 | 5′-GCATCCGTGCTGGCgCTCAACGCCTCACC-3′ | - |
| P166A_#2 | 5′-GGTGAGGCGTTGAGGGCCAGCACGGATGC-3′ | - |
| Δmyc_#1 | 5′-CATATGTATATCTCCTTCTTAAAGTTAAACAAAAT-3′ | - |
| Δmyc_#2 | 5′-AGTGGAATACTGACGCGCTGGCGAC-3′ | - |
| 133-170_#2 | 5′-ATGATGTCTGAAAAGGGTTATCGCATTGATTATGC-3′ | - |

Restriction enzyme recognition sites are underlined. The bold sequences encode myc-tag. The mutated codons are indicated as boxed nucleotides.
